# 2348 Lafora disease premature termination codons (PTCs) are likely candidates for suppression by aminoglycosides

**DOI:** 10.1017/cts.2018.90

**Published:** 2018-11-21

**Authors:** Zoe R. Simmons, Amanda Sherwood, Selena Li, Sylvie Garneau-Tsodikova, Matthew Gentry

**Affiliations:** 1 Center for Clinical and Translational Science, University of Kentucky; 2 Department of Molecular Medicine and Biochemistry, University of Kentucky, Lexington, KY, USA; 3 Department of Pharmacology and Nutritional Sciences, University of Kentucky, Lexington, KY, USA

## Abstract

OBJECTIVES/SPECIFIC AIMS: A small molecule therapy is within reach to treat a molecular mechanism known to result in thousands of fatal diseases. For 10% of patients with a genetic disease, a nonsense/STOP mutation/premature termination codon (PTC) is the underlying cause of their malady. PTCs prematurely stop protein synthesis and yield truncated proteins. Truncated proteins typically provide little to no proper function or activity and are rapidly degraded; thus, disease is imminent. Recent work has demonstrated that small molecules including aminoglycosides can cause the ribosome to readthrough these PTCs. Thus, PTC readthrough with small molecules is a very attractive approach for treating diseases caused by PTCs. Small molecules that promote readthrough act on the ribosome and induce a ribosomal conformational change. In this conformation, the PTC is not recognized by the translational machinery and an amino acid is incorporated into the growing peptide chain, thus protein synthesis continues and does not stop. The use of a single small molecule to readthrough various PTC mutations has been repeatedly effective for in vitro studies and some of these have progressed to clinical trials. Although there has been success in defining these small molecules, the field has discovered that every PTC is unique and likely requires a different small molecule. Thus, developing a cell culture model to test read-through of Lafora PTCs and the functionality of the protein product is the first step to developing a readthrough therapy for a LD. METHODS/STUDY POPULATION: Method for in vitro quantification of readthrough: 24 hours before transfection, HEK293 cells were split in 6-well plates. On the following day, approximately 60% confluence, the cells were transiently transfected with the WT or PTC mutated constructs using Polyethylenimine HCl MAX. Cells were transfected with a total amount of 0.35 μg DNA/well and 2 μl Polyethylenimine HCl MAX/well. Four hours later, the transfection medium was removed and replaced with fresh medium, without streptomycin and penicillin. The fresh media contained gentamicin diluted to the indicated concentration per well. Fresh gentamicin-containing medium was replaced after 24 hours. After 48 hours, lysates were collected in 100 μL mRIPA supplemented with protease inhibitors for each construct. The lysates were run on a western blot and the *N*-terminal was probed with anti-FLAG. A malachite green phosphatase assay to measure inorganic phosphate release from phospho-glucans, that is glycogen or LBs. Glycogen is used in this laforin bioassay as the biologically relevant substrate in order to determine the specific activity of the readthrough products. All reactions are incubated for 40 minute the absorbance is measured at 620 nm and the pmoles of phosphate released/min/nmol protein was calculated using a standard curve. RESULTS/ANTICIPATED RESULTS: HEK293 cells were transfected with MeCP2 R241X, laforin R241X, or laforin WT NT-FLAG construct, treated with different concentrations of gentamicin for 48 hours, and laforin levels were assessed by Western analysis with anti-FLAG. HEK293 cells were transfected with WT laforin or a laforin PTC CT-FLAG construct, treated with different concentrations of gentamicin for 48 hours, and laforin levels were assessed by Western analysis with anti-FLAG. B. Quantification of read-through for PTC experiments. *p-value≤0.001. #*p*-value≤0.001. Schematic of laforin bioassay. The assay has been performed with human and mouse tissue as well as cultured cells. B. Laforin bioassay results using laforin from PTC experiment. **p-value≤0.001. *p-value≤0.01. DISCUSSION/SIGNIFICANCE OF IMPACT: Our results suggest that gentamicin is not only responsible for inducing readthrough of the PTC mutations, but also for promoting translation of fully functional laforin. Therefore, our in vitro system for the analysis of PTC readthrough of laforin will be useful for determining which PTC mutations are suppressible with gentamicin or other small molecules, in what quantities laforin is recovered from PTC mutations, and if the protein products possess the appropriate enzymatic function.

